# Correction: Unraveling variation on the profile aroma compounds of strong aroma type of Baijiu in different regions by molecular matrix analysis and olfactory analysis

**DOI:** 10.1039/d1ra90161c

**Published:** 2021-10-29

**Authors:** Jiaxin Hong, Junshan Wang, Chunsheng Zhang, Zhigang Zhao, Wenjing Tian, Yashuai Wu, Hao Chen, Dongrui Zhao, Jinyuan Sun

**Affiliations:** Key Laboratory of Brewing Molecular Engineering of China Light Industry, Beijing Technology and Business University Beijing 100048 China zdrui6789@sina.com +86-10-68984890 +86-10-68988715; Chengde Qianlongzui Distillery Company Hebei 100048 China; Food and Biological Engineering, Beijing Vocational College of Agriculture Beijing 102442 China

## Abstract

Correction for ‘Unraveling variation on the profile aroma compounds of strong aroma type of Baijiu in different regions by molecular matrix analysis and olfactory analysis’ by Jiaxin Hong *et al.*, *RSC Adv.*, 2021, **11**, 33511–33521. DOI: 10.1039/D1RA06073B.

The authors regret the omission of a funding acknowledgement in the original article. This acknowledgement is given below.

Dongrui Zhao would like to acknowledge the National Natural Science Foundation of China (32001826).

The Royal Society of Chemistry apologises for these errors and any consequent inconvenience to authors and readers.

## Supplementary Material

